# Cardiometabolic Risk Profiles in Patients With Impaired Fasting Glucose and/or Hemoglobin A1c 5.7% to 6.4%: Evidence for a Gradient According to Diagnostic Criteria: The PREDAPS Study

**DOI:** 10.1097/MD.0000000000001935

**Published:** 2015-11-06

**Authors:** Carolina Giráldez-García, F. Javier Sangrós, Alicia Díaz-Redondo, Josep Franch-Nadal, Rosario Serrano, Javier Díez, Pilar Buil-Cosiales, F. Javier García-Soidán, Sara Artola, Patxi Ezkurra, Lourdes Carrillo, J. Manuel Millaruelo, Mateu Seguí, Juan Martínez-Candela, Pedro Muñoz, Albert Goday, Enrique Regidor

**Affiliations:** From the Department of Preventive Medicine and Public Health (History of Science), Universidad Complutense de Madrid (CG-G, ER), Instituto de Investigación Sanitaria del Hospital Clinico San Carlos (IdISSC), Madrid (CG-G, ER), Torrero-La Paz Health Center, Zaragoza (FJS, JMM), Department of Preventive Medicine and Quality Management, Hospital General Universitario Gregorio Marañón, Madrid (AD-R), Raval-Sud Primary Care Team, Barcelona (JF-N), Martín de Vargas Health Center, Madrid (RS), Tafalla Health Center, Navarra (JD), Azpilagaña Primary Care Team, Navarra (PB-C), Porriño Health Center, Pontevedra (JG-S), Hereza Health Center, Madrid (SA), Zumaia Health Center, Guipúzcua (PE), La Victoria de Acentejo Health Center, Santa Cruz de Tenerife (LC), Es Castell Basic Health Unit, Islas Baleares (MS), Yecla Health Center, Murcia (JM-C), Family and Community Medicine Teaching Unit, Cantabria (PM), Endocrinology and Nutrition Department, Del Mar Hospital, Barcelona (AG); and Consortium for Biomedical Research in Epidemiology and Public Health (CIBER en Epidemiología y Salud Pública—CIBERESP), Madrid, Spain (ER).

## Abstract

It has been suggested that the early detection of individuals with prediabetes can help prevent cardiovascular diseases. The purpose of the current study was to examine the cardiometabolic risk profile in patients with prediabetes according to fasting plasma glucose (FPG) and/or hemoglobin A1c (HbA1c) criteria.

Cross-sectional analysis from the 2022 patients in the Cohort study in Primary Health Care on the Evolution of Patients with Prediabetes (PREDAPS Study) was developed. Four glycemic status groups were defined based on American Diabetes Association criteria. Information about cardiovascular risk factors–body mass index, waist circumference, blood pressure, cholesterol, triglycerides, uric acid, gamma-glutamyltransferase, glomerular filtration–and metabolic syndrome components were analyzed. Mean values of clinical and biochemical characteristics and frequencies of metabolic syndrome were estimated adjusting by age, sex, educational level, and family history of diabetes.

A linear trend (*P* < 0.001) was observed in most of the cardiovascular risk factors and in all components of metabolic syndrome. Normoglycemic individuals had the best values, individuals with both criteria of prediabetes had the worst, and individuals with only one–HbA1c or FPG–criterion had an intermediate position. Metabolic syndrome was present in 15.0% (95% confidence interval: 12.6–17.4), 59.5% (54.0–64.9), 62.0% (56.0–68.0), and 76.2% (72.8–79.6) of individuals classified in normoglycemia, isolated HbA1c, isolated FPG, and both criteria groups, respectively.

In conclusion, individuals with prediabetes, especially those with both criteria, have worse cardiometabolic risk profile than normoglycemic individuals. These results suggest the need to use both criteria in the clinical practice to identify those individuals with the highest cardiovascular risk, in order to offer them special attention with intensive lifestyle intervention programs.

## INTRODUCTION

Prediabetes is a term used to designate a condition associated with an increased risk of developing diabetes, which includes an intermediate group of individuals whose blood glucose levels are higher than normal, but do not reach the cut-point values for diagnosing diabetes. The American Diabetes Association (ADA) recognize the following value ranges to identify individuals with prediabetes: fasting plasma glucose (FPG) levels from 100 mg/dL (5.6 mmol/L) to 125 mg/dL (6.9 mmol/L), defined as impaired fasting glucose (IFG); 2-hour values in the oral glucose tolerance test from 140 mg/dL (7.8 mmol/L) to 199 mg/dL (11.0 mmol/L), defined as impaired glucose tolerance (IGT); and hemoglobin A1c (HbA1c) from 5.7% to 6.4%.^1^

In addition to the high risk of diabetes, individuals with prediabetes are at increased risk of developing cardiovascular diseases.^2,3^ Also, a large percentage of these risk individuals have the metabolic syndrome,^4,5^ a cluster of cardiovascular risk factors that also has been associated with diabetes and cardiovascular diseases,^6^ and has been pointed as a clinical indicator of macro and microvascular diabetes complications.^7^

There is evidence that each of the glycemic measures used to identify prediabetes represents a different domain of glucose metabolism. While FPG reflects basal disglycemia, HbA1c reflects chronic exposure to basal and postprandial hyperglycemia.^8^ Also, it has been pointed that some characteristics, such as sex, race, and age of individuals with prediabetes vary by glycemic measure.^9^ Thus, there may be differences in the cardiometabolic risk profiles of individuals according to the glycemic measure used to evaluate the presence of prediabetes.

Several studies have been performed in nondiabetic individuals, searching for differences in cardiometabolic risk factors among IFG, IGT, and IFG-IGT groups.^10–13^ Comparing groups formed on the basis of HbA1c and FPG criteria, however, are sparse.^14^ Therefore, our aim was to examine the clinical and biochemical characteristics, and the prevalence of metabolic syndrome components in patients grouped into 4 glycemic statuses according to HbA1c and FPG criteria of prediabetes.

## METHODS

### Study Design and Populations

The Cohort study in Primary Health Care on the Evolution of Patients with Prediabetes [*Estudio de Cohortes en Atención Primaria sobre la evolución de sujetos con prediabetes* (PREDAPS study)] is a follow-up study of a cohort of patients with prediabetes and another cohort of patients without glucose metabolism disorders. Complete information about the design and methods of PREDAPS study have been previously described.^15^ In summary, the study is being conducted by general practitioners distributed across Spain, in the context of their routine clinical practice. At the baseline stage, in 2012, patients aged between 30 and 74 years old, whom consecutively sought medical attention for any reason, were invited to participate in the study. Patients were excluded if they had diabetes, terminal disease, pregnancy, surgery, or hospital admission in the previous 3 months at study entry, or any hematologic disease, which could alter HbA1c values. A total of 2022 patients gave their written informed consent for participation in this study. The study was classified by the Spanish Agency of Medicines and Medical Devices (*Agencia Española de Medicamentos y Productos Sanitarios*) as a Non-Interventional (Observational) Post-Authorization study, and the study protocol was approved by the Parc de Salut Mar Clinical Research Ethics Committee in Barcelona.

According to criteria for categories of increased risk for diabetes defined by ADA,^1^ patients were classified into 1 of 4 mutually exclusive groups of glycemic status on the basis of plasma levels of HbA1c and FPG: 1) Normoglycemia group (HbA1c < 5.7% and FPG < 100 mg/dL), 2) Isolated HbA1c group (HbA1c 5.7%–6.4% and FPG < 100 mg/dL), 3) Isolated FPG group (HbA1c < 5.7% and FPG 100–125 mg/dL), and 4) Both criteria group (HbA1c 5.7%–6.4% and FPG 100–125 mg/dL).

### Measurements

Information about sociodemographic characteristics–age, sex, educational level–and family history of diabetes was obtained through questionnaire at baseline for all individuals. The patients underwent physical examination to measure anthropometric parameters–height, weight, and waist circumference–and blood pressure–3 readings–. Also, a blood sample was obtained to determine plasma levels of the following biochemical parameters: total cholesterol, high-density lipoprotein cholesterol (HDL cholesterol), triglycerides, uric acid, gamma glutamyltransferase, hemoglobin, creatinine, FPG, and HbA1c.

In this analysis, body mass index was calculated as weight in kilograms divided by the square of height in meters. Systolic and diastolic blood pressures were estimated by averaging of the 3 readings taken. Low-density lipoprotein cholesterol (LDL cholesterol) was estimated by Friedewald equation.^16^ Glomerular filtration was calculated using the abbreviated Modification of Diet in Renal Disease (MDRD) equation.^17^

Metabolic syndrome was defined according to the 2009 harmonizing statement,^18^ although we also considered a value of HbA1c ≥5.7% as criteria of elevated glycemia. Then, the components of metabolic syndrome considered in this analysis were defined as follows: elevated waist circumference (≥102 cm in men or ≥88 cm in women), elevated triglycerides (≥150 mg/dL or drug treatment for elevated triglycerides), reduced HDL cholesterol (<40 mg/dL in men or <50 mg/dL in women, or drug treatment for reduced HDL cholesterol), elevated blood pressure (systolic blood pressure ≥130 mm Hg, or diastolic blood pressure ≥85 mm Hg, or antihipertensive drug treatment in patients with history of hypertension), and elevated glycemia (HbA1c ≥ 5.7%, or FPG ≥ 100 mg/dL, or drug treatment for elevated glycemia). Each measure involved in metabolic syndrome definition was transformed into binary variables to identify the presence or absence of each component, according to cut points listed above. The presence of at least 3 of 5 components was considered as a diagnosis of metabolic syndrome.

### Statistical Analysis

Distribution of sociodemographic variables and family history of diabetes according to study groups was compared using the χ^2^test. Then, for each glycemic status group, the mean values of clinical and biochemical characteristics—adjusted for age, sex, educational level, and family history of diabetes—were estimated, and the differences in these values with respect to the normoglycemia group were calculated. These analyzes were performed by analysis of covariance. The adjusted frequencies of metabolic syndrome and its components were estimated using probit models. Finally, to know the number of components in each group, the prevalence of patients with 0, 1, 2, 3, and 4 components of the metabolic syndrome was estimated excluding the elevated glycemia component. Polynomial contrasts were used for testing linear trends across groups. *P* values <0.05 were considered as statistically significant. Statistical analysis was performed using IBM SPSS Statistics for Windows version 19 (IBM Corp, Armonk, NY).

## RESULTS

A total of 838 participants (41.4%) were classified into normoglycemia group, 316 (15.6%) into isolated HbA1c group, 254 (12.6%) into isolated FPG group, and 614 (30.4%) into both criteria—HbA1c 5.7% to 6.4% and FPG 100 to 125 mg/dL—group.

Sociodemographic characteristics and family history of diabetes according to the glycemic status are presented in Table 1. Patients with both criteria of prediabetes were the oldest, and they had the highest proportion of low educational level and family history of diabetes. The proportion of men was greater in the isolated FPG group, whereas the proportion of women was greater in the isolated HbA1c group.

**TABLE 1:**
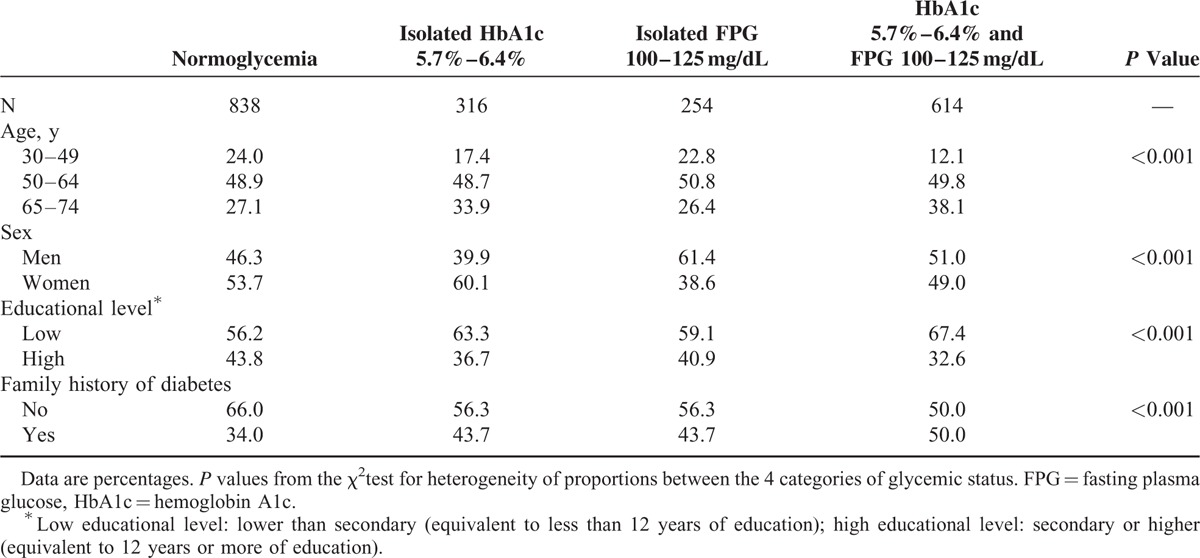
Distribution of Sociodemographic Characteristics and Family History of Diabetes by Glycemic Status

Means of clinical and biochemical characteristics adjusted by age, sex, educational level, and family history of diabetes are shown in Table 2 and mean differences with respect to normoglycemia group are presented in Table 3. A linear trend was observed in body mass index, waist circumference, systolic and diastolic blood pressure, HDL cholesterol, triglycerides, uric acid, and gamma glutamyltransferase values. The mean differences in these parameters showed the highest absolute value in the group with both criteria of prediabetes. In total cholesterol, LDL cholesterol, hemoglobin, and glomerular filtration parameters, no differences were found.

**TABLE 2:**
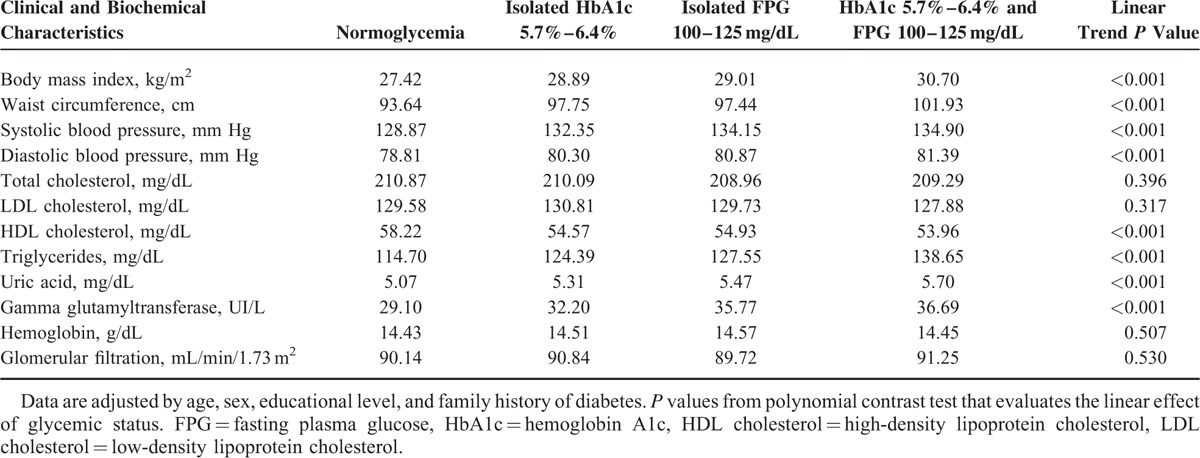
Means of Clinical and Biochemical Characteristics by Glycemic Status, and *P* Values for Linear Trend

**TABLE 3:**
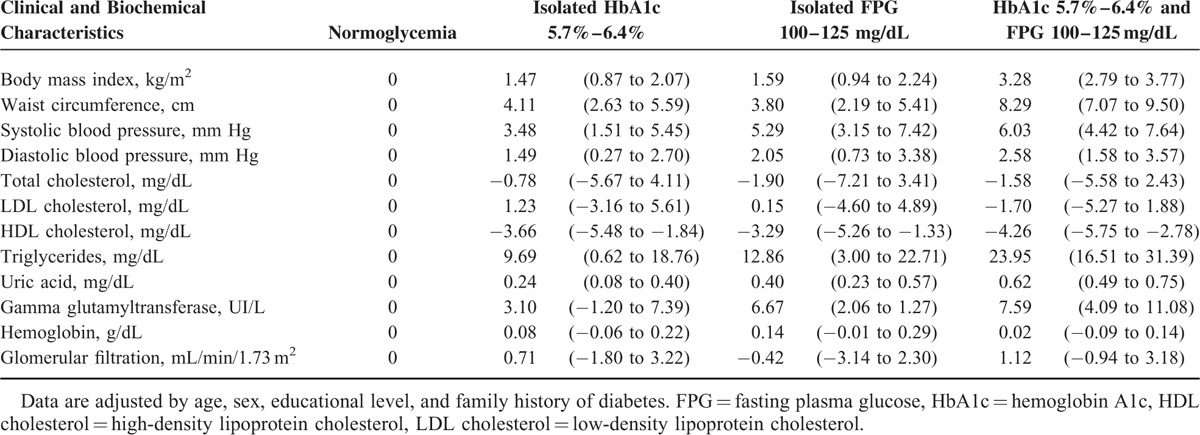
Adjusted Mean Differences (95% Confidence Intervals) in Clinical and Biochemical Characteristics According to Glycemic Status

Frequencies of each of the metabolic syndrome components, adjusted for age, sex, educational level, and family history of diabetes, are shown for the 4 glycemic status groups in Table 4. The most prevalent component in all groups was elevated blood pressure, followed by elevated waist circumference. A progressive increase of frequency in all components according to glycemic status was observed, such that the lowest frequency was observed in the normoglycemia group, followed by isolated HbA1c group, isolated FPG group, and the highest in the both criteria group. The frequency of the metabolic syndrome as a whole was 15.0% in patients with normoglycemia, 59.5% in patients with isolated HbA1c criterion, 62.0% in patients with isolated FPG criterion, and 76.2% in patients with both criteria of prediabetes. Because of the low prevalence in normoglycemic patients with respect to the other groups, the trend in the magnitude of the prevalence over the 4 groups was deviated from linearity.

**TABLE 4:**
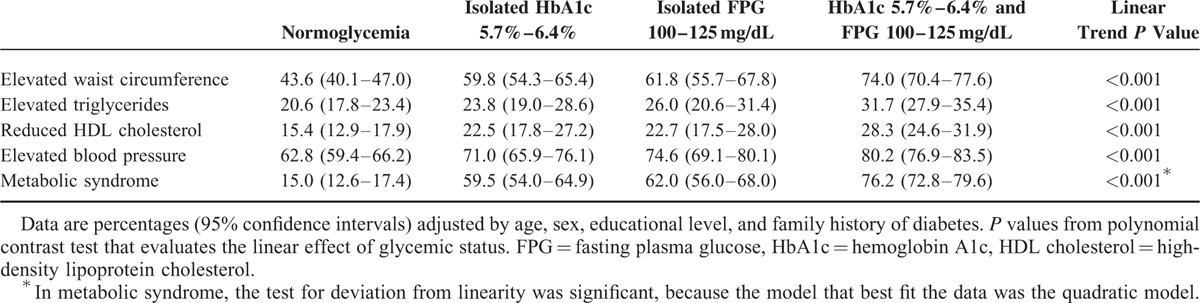
Frequencies of Metabolic Syndrome Components by Glycemic Status, and *P* Values for Linear Trend

**FIGURE 1:**
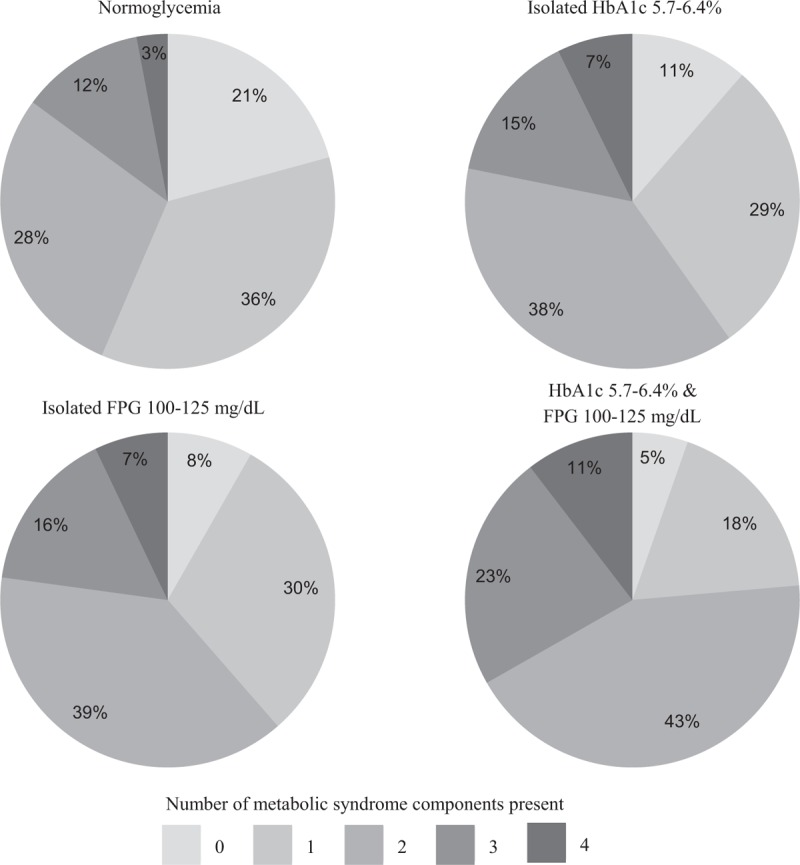
Percentages of each number of metabolic syndrome components present (excluding the “elevated plasma glucose” component) in each glycemic status group.

The distribution of the number of components present—excluding elevated glycemia component—for each glycemic status group is showed in the Figure 1. It can be seen that the percentage of individuals with either 0 or 1 component is greater in the normoglycemia group, whereas the percentage of individuals with 2, 3, or 4 components is greater in the group with both criteria of prediabetes. The percentages of patients who did not meet any component of metabolic syndrome were 21% in individuals with normoglycemia, 11% in those with isolated HbA1c criterion, 8% in those with isolated FPG criterion, and 5% in those with both criteria. On the other end of the distribution, patients who meet 4 components, the percentages were 3%, 7%, 7%, and 11%, respectively.

## DISCUSSION

Individuals with prediabetes defined by both HbA1c and FPG criteria had the worst values in clinical and biochemical characteristics related to increase cardiovascular risk, and the highest frequency of metabolic syndrome. On the other hand, individuals with prediabetes defined by only one—HbA1c or FPG—criterion were at an intermediate cardiometabolic risk profile, showing a more favorable profile than individuals with 2 criteria, but less favorable than individuals without any criteria of prediabetes.

Results of comparisons of the 4 glycemic status groups show that there are differences among groups in some of characteristics examined. It is worth mentioning, differences in sex distribution observed in prediabetic groups, where the proportion of men was greater in the isolated FPG group and the proportion of women was greater in the isolated HbA1c group. Similar finding can be observed in other studies with prediabetic groups defined by HbA1c and FPG criteria.^19,20^ Also, Lipska et al^21^ have been pointed that women were more likely to be identified with dysglycemia by HbA1c than FPG.

Studies comparing cardiometabolic risk measures in prediabetes groups defined by FPG and IGT criteria have also found in categories with any isolated criterion, an intermediate risk profile between normoglycemic status—low risk—and the combination of 2 criteria of prediabetes—high risk.^10,11^ Moreover, other studies based on nondiabetic individuals, which have considered HbA1c and FPG as prediabetes criteria, had observed higher levels of some cardiovascular risk factors in individuals with prediabetes by both criteria.^19,20^ By contrast, no differences in cardiometabolic risk profiles were observed by Marini et al^14^ when they compared 3 groups of individuals with prediabetes based on the same criteria. The fact that having at least 1 cardiometabolic risk factor was a criterion to include participants in their study could explain this inconsistency, because their study groups were more homogeneous respect to baseline risk.

There is sufficient evidence that metabolic syndrome is strongly related with hyperglycemia.^4,5,22^ As expected, we found a high percentage of metabolic syndrome in individuals with prediabetes. A higher prevalence of this syndrome in individuals with both criteria of prediabetes than in those with only one criterion has been reported.^5,10^ In our study, apart from the frequency of metabolic syndrome as a whole, the percentage of individuals in normoglycemia group who had no metabolic syndrome components present was 4 times higher than the corresponding percentage in both criteria group. While the percentage of individuals in normoglycemia group who met the 4 metabolic syndrome components was less (approximately half) than the corresponding percentage of individuals in isolated criterion groups, and even less (approximately one-third) compared with the same in the both criteria group. This is consistent with the other findings of this study, the cardiometabolic risk increases according to the number of prediabetes criteria present increases.

The worst risk profile observed in individuals with both criteria of prediabetes could be explained by the differences in the role of FPG or HbA1c to detect glucose metabolism disorders. If each measure represents a different pathophysiologic mechanism, it can be expected that individuals with both criteria of prediabetes have a higher risk than those with only one criterion.

Compared with individuals in the isolated FPG group, it is noteworthy that those in the isolated HbA1c group had slightly lower values in the most of clinical and biochemical parameters related to cardiovascular risk. In this sense, Heianza et al^20^ have pointed a similar finding: individuals diagnosed by isolated HbA1c criterion were more likely to have lower values in body mass index, blood pressure, and serum concentrations of triglyceride, HDL cholesterol, uric acid, and gamma glutamyltransferase. The above results could be indicative of a lower cardiovascular risk profile in individuals with isolated HbA1c criterion at diagnosis of prediabetes. We, however, must not overlook that more favorable waist circumference and HDL cholesterol values were observed in the isolated FPG group. Moreover, there are discrepancies in findings across studies. For example, Saukkoken et al^23^ reported higher mean values of body mass index and triglycerides in the isolated HbA1c group compared with isolated FPG group, and not differences in blood pressure and waist circumference between both groups. And other authors have also reported contradictory results, for some parameters slightly lower values were observed in HbA1c group, and for others in FPG group.^14,19^

In a recent study conducted in middle-aged Caucasian-Europeans, it can be observed that the subjects classified as prediabetic using HbA1c criterion has lower body mass index, waist circumference, and triglycerides levels and higher HDL cholesterol than those subjects classified using FPG criterion.^24^ In this study, when we analyzed waist circumference and HDL cholesterol parameters using metabolic syndrome components cut points, we also observed that frequencies of these criteria were lower in isolated HbA1c group than isolated FPG group. The same has been seen for elevated triglycerides and elevated blood pressure criteria, and metabolic syndrome as a whole. This finding is consistent with those studies on prevalence of metabolic syndrome comparing HbA1c criterion of elevated glycemia with FPG criterion, where the prevalence was lower when alone HbA1c criterion was considered to determine the presence of syndrome.^25–27^ However, in the only one study that shows results of metabolic syndrome components, the frequencies of elevated waist circumference and reduced HDL cholesterol were higher in the group based on HbA1c criterion than in the group based on FPG.^25^ On the other hand, higher FPG levels rather than higher HbA1c levels have been more strongly associated with an increased risk for development of hypertension at 5 years among Japanese.^28^

Then, several hypotheses can be proposed about the presence of isolated HbA1c criterion at prediabetes diagnosis. First of all, it identifies individuals with a slightly lower metabolic risk status compared with those detected by isolated FPG criterion. Second, it identifies individuals with a risk status related to a very early stage of cardiovascular disease. And finally, it cannot be ruled out that isolated HbA1c criterion is more specific to identify those characteristics, which are more strongly related to cardiovascular diseases, such as low HDL cholesterol and high waist circumference.

This study is being carried out in routine clinical practice and includes a large number of individuals across nationwide, which are being followed annually. Despite this strength, all patients studied are primary health care users, and the frequency of cardiometabolic risk factors could be different from other settings or from the general population. So care should be taken in generalizing these results.

This study had some limitations. First, patients were classified into groups based on single measurements of HbA1c and FPG, and so we cannot rule out the possibility of misclassification bias because of potential problems with intraindividual variability of glycemia measures.^29^ Second, a level of HbA1c ≥ 5.7% was considered as an additional criterion of elevated glycemia component of metabolic syndrome, which is not included in current definitions of this syndrome. We, however, calculated the frequencies of metabolic syndrome excluding the elevated glycemia component (data not shown) and the results were very similar to those observed when this component was included. This finding support the use of the HbA1c levels in range of prediabetes as an additional measure to screen glycemic component of metabolic syndrome, an issue that has been pointed by some authors.^25,30^

In conclusion, individuals with prediabetes have a worse cardiometabolic risk profile than normoglycemic individuals, and those with both criteria of prediabetes have the worst risk profile. These results suggest the need to use both criteria in the clinical practice to identify those individuals with the highest cardiovascular risk in order to offer them special attention with intensive lifestyle intervention programs. Some of our findings suggest that individuals with isolated HbA1c criterion at diagnosis of prediabetes might have a slightly lower cardiometabolic risk than those with isolated FPG criterion, but further studies are needed on this topic.
